# Growth of all-carbon horizontally aligned single-walled carbon nanotubes nucleated from fullerene-based structures

**DOI:** 10.1186/1556-276X-8-265

**Published:** 2013-06-06

**Authors:** Imad Ibrahim, Yang Zhang, Alexey Popov, Lothar Dunsch, Bernd Büchner, Gianaurelio Cuniberti, Mark H Rümmeli

**Affiliations:** 1 IFW-Dresden e.V., PF 270116, Dresden 01171, Germany; 2 Institute for Materials Science and Max Bergmann Center of Biomaterials, Technische Universität Dresden, Dresden 01062, Germany; 3 Department of Physics, Technische Universität Dresden, Dresden 01062, Germany

**Keywords:** Horizontally aligned SWCNT, CVD, Catalyst-free SWCNT, Fullerene nucleate CNT

## Abstract

All-carbon single-walled carbon nanotubes (SWCNTs) were successfully synthesized, nucleated using a fullerene derivative. A systematic investigation into the initial preparation of C_60_ fullerenes as growth nucleators for the SWCNTs was conducted. Enhancement in the yield of the produced SWCNT has been achieved with exploring different dispersing media for the fullerenes, the period, and environment of the initial thermal treatment of the fullerenes in addition to the use of different fullerene-based structures. The systematic studies significantly advance our understanding of the growth of the all-carbon catalyst-free single-walled carbon nanotubes. Field-effect transistors were fabricated using the catalyst-free SWCNT and then electrically characterized, showing current capacity as high as the well-studied catalyst-assisted nanotubes.

## Background

Enormous efforts have been invested towards the realization of single-walled carbon nanotube (SWCNT)-based products due to their extraordinary properties [1,2]. One of the more attractive potential applications of these exciting nanostructures is as a building block for nanoelectronics. To this end, individual or parallel-aligned SWCNTs with tunable yield are important [3,4]. For such applications, however, the reproducible control of the nanotubes’ spatial orientation and chiral management still require further development [5]. Some success has been achieved regarding the controlled fabrication of well-oriented nanotubes, especially when directly fabricating aligned tubes using chemical vapor deposition (CVD) [6,7]. Usually though, a catalyst particle (mostly metal catalyst particles) are used to nucleate the growth of the nanotubes, and this has a drawback since the catalyst particles may diffuse into the substrate or tube and thus affect their intrinsic properties or that of a device built around them [8,9]. Therefore, the synthesis of a catalyst-free-aligned SWCNT is very attractive. Different all-carbon routes have been developed, for example, using diamonds as open-ended SWNT and fullerenes as SWCNT nucleators [10-12]. However, the yield of the grown tubes is generally low. Moreover, this remains a very limited understanding of all-carbon SWCNT growth.

In this study, we systematically investigate aspects related to yield from metal-free horizontally oriented SWCNTs nucleated from pristine C_60_ fullerenes and exohedrally functionalized C_60_F_18_ fullerenes. Aside from direct comparisons between the two types of fullerenes, we also investigate the role of the dispersing solution and pretreatment steps to functionalize and activate them prior to CVD growth.

## Methods

Nominal amounts of fullerene derivatives (C_60_ and C_60_F_18_), which will later serve as nanotube nucleators, were homogenously dispersed independently in toluene, acetone, and ethanol by overnight ultrasonication. Single crystal quartz substrates (10 × 10 × 0.5 mm, angle cut 38° 00’, single side polished from Hoffman Materials, LLC, Carlisle PA, USA), were initially subjected to thermal annealing in air at 750°C for 15 min prior to the chemical vapor deposition (CVD) reaction for nanotube growth. This results in a smoother surface which helps provide higher yields [7]. The initial fullerenes were then placed on the quartz substrate prior to these treatments by drop coating the dispersed fullerenes. The deposited fullerenes are opened (to form open caps that serve as nucleation centers) and then activated by functionalization. These processes are accomplished by first heating the loaded substrates in various environments (air, synthetic air, Ar or H_2_) for different periods (10 to 120 min) at temperatures between 400°C and 500°C in a 1-in purpose-built horizontal tube furnace. Thereafter, the activation is achieved by heating the samples at 900°C in water vapor (0.17 standard liter per minute (SLPM) Ar bubbled through water) for 2 min and then heating in hydrogen (0.75 SLPM) for the next 3 min. Later, the CVD reaction was performed in a gaseous environment of hydrogen (4.5 SLPM), Ar (0.2 SLPM), and Ar (0.32 SLPM) bubbled through ethanol, keeping the temperature stable at 900°C for 20 min.

Atomic force microscopy (Digital Instruments NanoScope IIIa, Veeco, Plainview, NY, USA) operating in the tapping mode was employed to characterize the fullerenes after the different treatment steps and also assess the yield and diameter of the nanotubes after CVD growth. The length and alignment of the CNTs were determined using a scanning electron microscope (SEM; NOVA 200 NanoSEM, FEI, Hillsboro, OR, USA; with typical acceleration voltage of 2 to 3 kV), while the type and quality of the grown tubes were estimated by transmission electron microscopy (a double Cs-Corrected JEM-2010 F, JEOL, Akishima-shi, Japan; using an acceleration voltage of 80 kV) and Raman spectroscopy (DXR SmartRaman Thermo Scientific, Waltham, MA, USA; λ = 533 nm). For the electrical measurements, a set of source-drain electrode pairs (10 nm Cr, 40 nm Au) in addition to the gate electrode (50 nm Al_2_O_3_, 10 nm Cr, 40 nm Au) were fabricated using standard e-beam lithography on the substrates where the nanotubes were as-grown.

## Results and discussion

**Figure 1:**
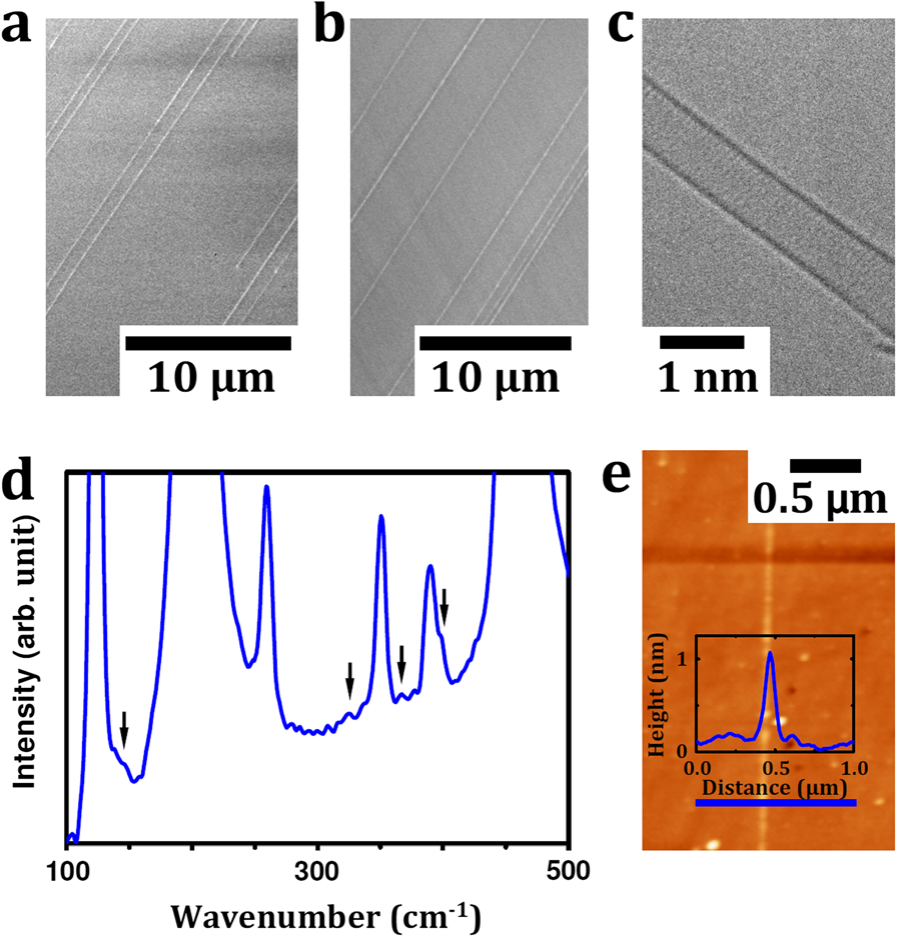
**Characterization of as-produced carbon nanotubes.** (**a** and **b**) Representative SEM images of CVD-grown horizontally aligned CNT nucleated from pristine fullerenes (C_60_) and exohedrally functionalized fluorofullerenes (C_60_F_18_), respectively. (**c**, **d,** and **e**) TEM micrograph, RBM of the Raman spectroscopy, and AFM image of catalyst-free SWCNT, respectively. An inset is the height profile of the nanotube shown in panel **e**.

**Figure 2:**
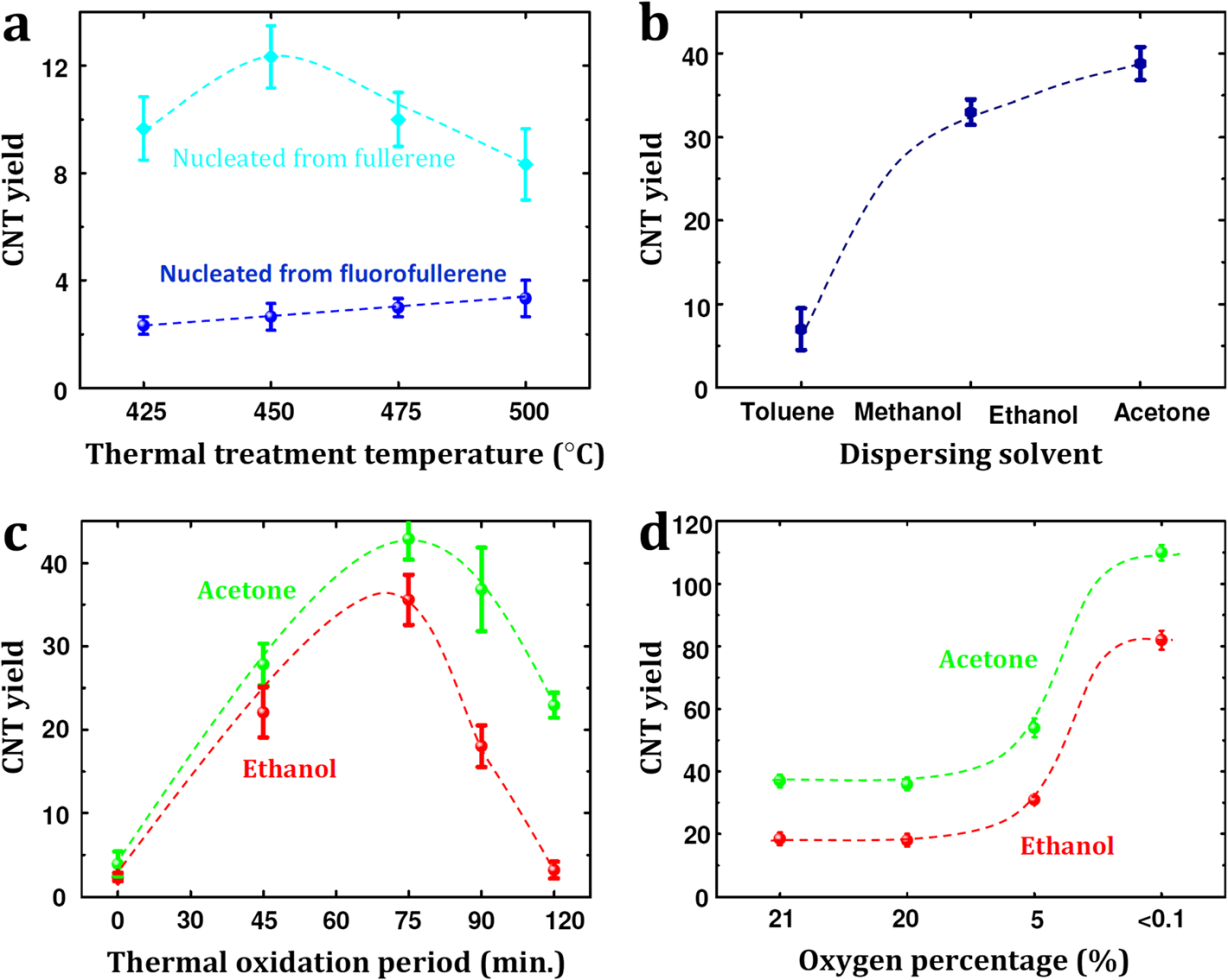
**Enhancement in the yield of the CVD-grown horizontally aligned SWCNT.** (**a**) Variation in the yield of the nanotubes grown from C_60_ and C_60_F_18_. Yield of carbon nanotube dependency on (**b**) initial fullerene dispersing media, (**c**) the thermal oxidation environment, and (**d**) thermal oxidation period.

**Figure 3:**
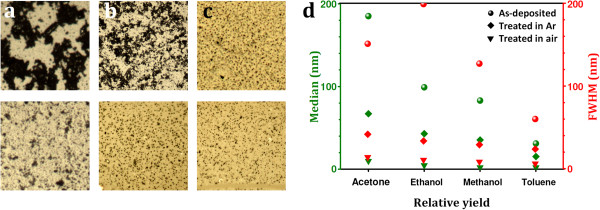
**Formation and size distribution of fullerene clusters formed on ST-cut quartz substrates.** By visible light microscopy (**a**) as-deposited, after pretreatment for 75 min in (**b**) air, and (**c**) Ar**.** The upper row shows clusters originally dispersed in acetone while the lower row shows those clusters originally dispersed in toluene. (**d**) Median and FWHM of the as-deposited and pretreated fullerene clusters.

**Figure 4:**
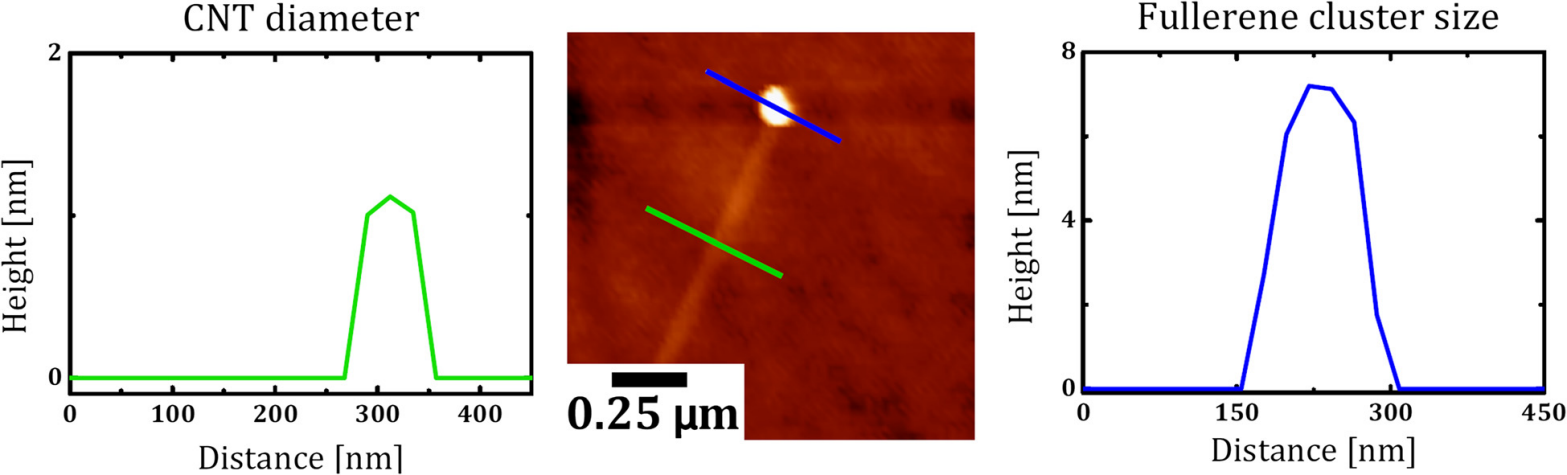
**Characterization of clusters at the end of the grown SWCNT.** Representative AFM images showing a globule at one end of the as-grown catalyst-free SWCNT along with the height profile of such globule feature to the right while the height profile of the grown CNT is to the left of the AFM image.

The treated and activated fullerene derivatives were successfully used to nucleate the single-walled carbon nanotubes grown by chemical vapor deposition. The CNT were grown on very smooth single crystal quartz substrates, as this has been shown to aid high yields of horizontally aligned SWCNTs [7]. The fullerene derivatives used in this study were pure C_60_ and fluorofullerene (C_60_F_18_). These two were compared by dispersing them first in toluene. The fluorofullerene is a C_60_ surrounded by 18 fluorine atoms on the cage of the C_60_ and provides a useful way to investigate the role of surface-functionalized C_60_ against non-functionalized C_60_. Typical SEM micrographs for the CNT nucleated from C_60_ and C_60_F_18_ are shown in Figure 1a,b, respectively. The grown CNTs are found to be single-walled, as shown in the representative transmission electron microscopy (TEM) micrograph (Figure 1c) and by the height profile extracted from the atomic force microscopy (AFM) characterization of the grown tubes (Figure 1e). Raman spectroscopy studies confirm the presence of single-walled tubes by the existence of radial breathing modes (RBM) in the spectra (Figure 1d), which are a well-known signature for SWCNT and are frequently used to estimate the diameter of the investigated nanotubes [13]. The grown SWCNT diameter distribution is in the range between 0.7 and 1.5 nm, as estimated from the Raman spectroscopy. A higher yield was achieved when using C_60_F_18_ as nucleators as compared to pristine C_60_, as shown in the representative SEM images provided in Figures 1a,b and 2a. We argue that this is due to the dramatic elongation of carbon atom bonds adjacent to the fluorine atoms, which allows them to break more easily and hence make the formation of a spherical cap, which is appropriate for the tube nucleation and is more efficient than the use of pristine C_60_ in the initial pre-synthesis step [14]. The higher yield (number of nanotubes per unit area) of the grown tubes achieved with the C_60_F_18_ fullerenes is attractive on one side while otherwise on the other because such exohedrally functionalized fullerenes are difficult to produce in large quantities, which make them economically unattractive in practical terms. Hence, we now focus on efficient routes to growing CNT nucleated from pure C_60_ fullerenes. To do this, we explore the role of the dispersing medium. We do this by comparing the yield after dispersing the C_60_ in toluene, acetone, and ethanol. A dramatic increment in the tube yield can be observed when using acetone as the dispersing medium as seen in Figure 2b. The yield of the tubes grown from C_60_ dispersed in ethanol is less than found for the dispersion in acetone but better than that for toluene. The reasons for this are discussed later. We now turn to the influence of the pretreatment steps to open and activate the fullerenes prior to exposing them to the CVD growth reaction. We first look at the opening of the fullerenes. Different thermal pretreatment periods in air result in different yields. The CNT yield increases with pretreatment time to a maximum at around 75 min, after which the yield drops. This is because with excessive oxidation, most of the fullerene clusters are burnt away. Further enhancement in the grown CNT yield was also achieved by optimizing the oxygen environment. It was found that a gas mixture of Ar or H_2_ with oxygen contents <0.1% was best. The variation in the CNT yield due to the change in the thermal oxidation period is shown in Figure 2c while the effect of the thermal oxidation environment is provided in panel d. The thermal oxidation step is required to open up the fullerenes so as to provide hemispherical caps which would later serve as the nucleation sites for continued tube growth [12]. The oxidation process diminishes the fullerene cluster size, as shown in Figure 3, in which optical micrographs for the as-deposited and thermally treated fullerenes originally dispersed in acetone (upper row) and in toluene (lower row) are provided. Panel b of the same figure presents the size distribution and full width at half maximum of the formed fullerene clusters before and after treatment in different environments. The cluster sizes increase markedly for ethanol and then acetone. This trend is the same even for the thermally treated clusters. A clear correlation between cluster size and yield can be observed (Figure 2b) larger cluster sizes lead to larger SWCNT yields, and this explains the trend previously observed for yield variation with dispersion medium. The as-grown SWCNT on the host substrate were also investigated by employing AFM, which reveals that the diameter distribution of the nanotubes is in the range between 0.7 and 1.4 nm in good agreement with the TEM and Raman spectroscopy investigations. Often, we observed a globular-like feature at the end of a tube (see Figure 4). We assume these are the clusters from which a tube buds and grows from. The bulb heights are in the range between 2 and 10 nm and show no correlation to the SWCNT diameters.

**Figure 5:**
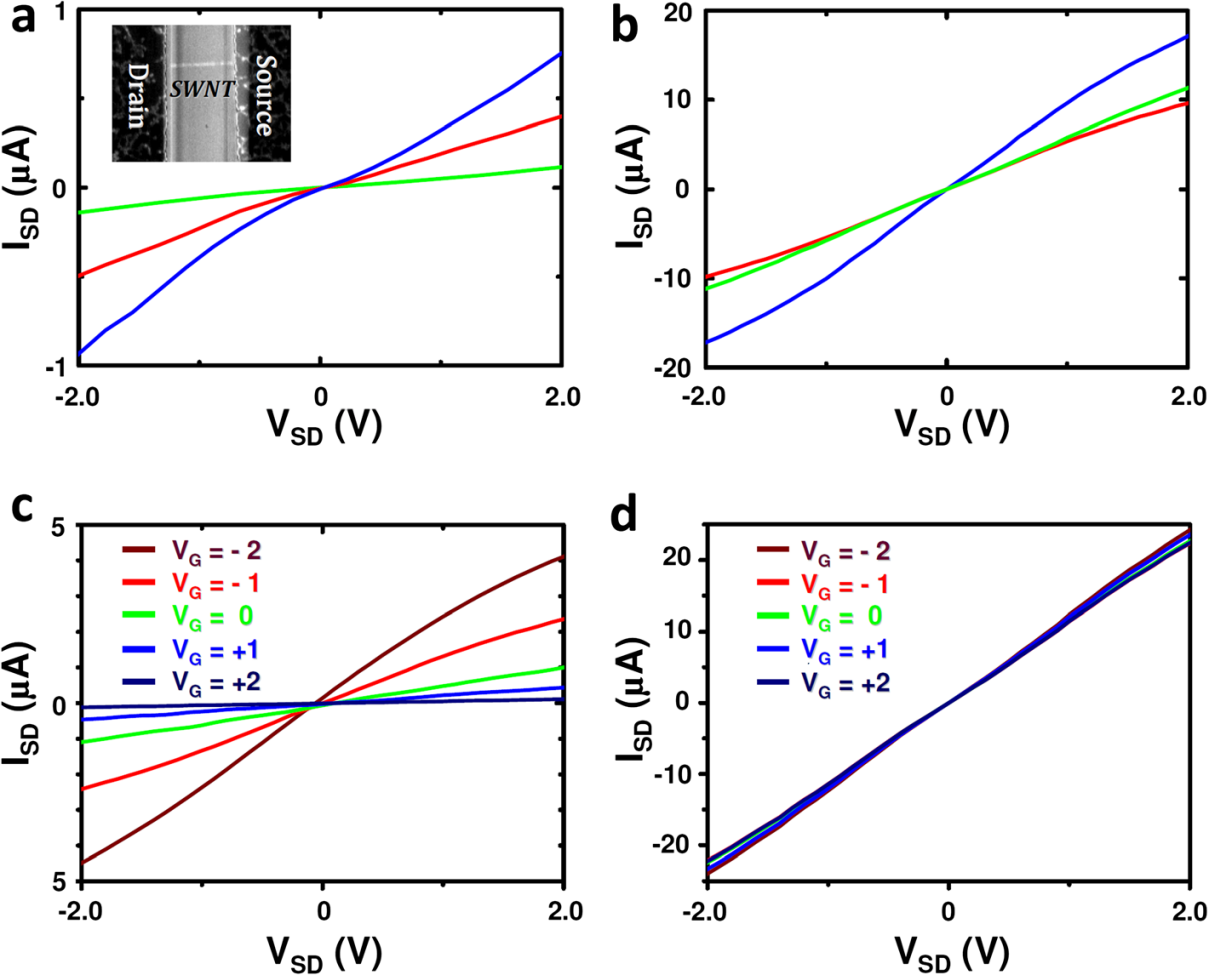
**Electrical characterization of the as-produced catalyst-free SWCNTs.** Two terminal IV characteristics of (**a**) semiconducting and (**b**) metallic SWCNTs. IV characteristic dependence on the gate voltage for (**c**) semiconducting and (**d**) metallic SWCNTs.

We also electrically characterized the as-grown SWCNT room temperature, firstly, by means of two terminal measurements and then they were gated and characterized once more. In the first step, source-drain electrode pairs were prepared by standard electron beam lithography. To characterize the tubes, a potential V_SD_ was applied across the electrodes and the current, with the V_SD_ measured. Typical two-terminal electrical characteristics from semiconducting nanotubes are shown in panel a of Figure 4. The electrical characteristics of the SWCNTs vary as they are dependent on the bandgap, which related to the nanotube chirality (diameter). Figure 5b shows typical IV characteristics of metallic nanotubes. The devices exhibit a resistance less than 150 kΩ. This high resistance is attributed to backscattering and contact effects, which results in I_SD_ saturation at high V_SD_[15]. Panels c and d of the same figure show the IV characteristics of semiconducting and metallic SWCNTs with applied gate voltages, respectively. The metallic nanotubes show no dependence on the gate voltage, as expected, the semiconducting nanotube behavior depends strongly on the applied gate voltage. They are found to conduct well at negative gate voltages while they are almost insulating at positive gate voltages. This indicates they are p-type semiconducting tubes [16].

## Conclusion

In summary, we have systematically investigated the pretreatment steps and growth of catalyst-free grown carbon nanotubes using opened and functionalized C_60_ and C_60_F_18_ as nucleation centers. The choice of dispersion agent is also important such that large clusters of initially deposited fullerenes lead to improved yields. Optimizing the thermal treatment steps to open and functionalize the fullerene clusters are also shown to improve the yield of the grown nanotubes. The as-synthesized tubes appear to be predominantly SWCNT. The high performance of the field-effect transistors fabricated using such catalyst-free SWCNTs make such tubes as promising candidates for future nanoelectronic applications.

## Competing interests

The authors declare that they have no competing interests.

## Authors’ contributions

IIYZ, AP, LD, BB, GC, and MR researched data for the article, contributed to the discussion of content, and reviewed and edited the manuscript before submission. All authors read and approved the final manuscript.

## References

[B1] Tans SJ Verschueren ARM Dekker C Room-temperature transistor based on a single carbon nanotube Nature 1998 8 49 52 10.1038/29954

[B2] Kang SJ Kocabas C Ozel Shim M Pimparkar N Alam MA Rotkin SV Rogers JA High-performance electronics using dense, perfectly aligned arrays of single-walled carbon nanotubes Nature Nanotech 2007 8 230 236 10.1038/nnano.2007.77 18654268

[B3] Ibrahim I Bachmatiuk A Warner JH Büchner B Cuniberti G Rümmeli MH CVD grown horizontally aligned single wall carbon nanotubes: Synthesis routes and growth mechanisms Small 2012 8 1973 1992 10.1002/smll.201102010 22619167

[B4] Kocabas C Hur S-H Gaur A Meit MA Shim M Rogers JA Guided growth of large-scale, horizontally aligned arrays of single-walled carbon nanotubes and their use in thin-film transistors Small 2005 8 1110 1116 10.1002/smll.200500120 17193404

[B5] Ishigami N Ago H Imamoto K Tsuji M Iakoubovskii K Minami N Crystal plane dependent growth of aligned single-walled carbon nanotubes on sapphire J Am Chem Soc 2008 8 9918 9924 10.1021/ja8024752 18597459

[B6] Krupke R Linden S Rapp M Hennrich F Thin films of metallic carbon nanotubes prepared by dielectrophoresis Adv Mater 2006 8 1468 1470 10.1002/adma.200600134

[B7] Ibrahim I Bachmatiuk A Börrnert F Blüher J Zhang S Wolff U Büchner B Cuniberti G Rümmeli MH Optimizing substrate surface and catalyst conditions for high yield chemical vapor deposition grown epitaxially aligned single-walled carbon nanotubes Carbon 2011 8 5029 5037 10.1016/j.carbon.2011.07.020

[B8] Brukh R Sae-Khow O Mitra S Stabilizing single-walled carbon nanotubes by removal of residual metal catalysts Chem Phys Lett 2008 8 149 152 10.1016/j.cplett.2008.05.026

[B9] Nel A Xia T Mädler L Li N Toxic potential of materials at the nanolevel Science 2006 8 622 627 10.1126/science.1114397 16456071

[B10] Takagi D Kobayashi Y Homma Y Carbon nanotube growth from diamond J Am Chem Soc 2009 8 6922 6923 10.1021/ja901295j 19405530

[B11] Yao Y Feng C Zhang J Liu Z “Cloning” of single-walled carbon nanotubes via open-end growth mechanism Nano Lett 2009 8 1673 1677 10.1021/nl900207v 19284730

[B12] Yu X Zhang J Choi W Choi J-Y Kim JM Gan L Liu Z Cap formation engineering: from opened C-60 to single-walled carbon nanotubes Nano Lett 2010 8 3343 3349 10.1021/nl1010178 20681626

[B13] Jorio A Saito R Hafner JH Lieber CM Hunter M McClure T Dresselhaus G Dresselhaus MS Structural (n, m) determination of isolated Single-walled carbon nanotubes by resonant raman scattering Phys Rev Lett 2001 8 1118 1121 10.1103/PhysRevLett.86.1118 11178024

[B14] Ibrahim I Bachmatiuk A Rümmeli MH Wolff U Popov A Boltalina O Büchner B Cuniberti G Growth of catalyst-assisted and catalyst-free horizontally aligned single wall carbon nanotubes Status Solidi B 2011 8 2467 2470 10.1002/pssb.201100073

[B15] Lazzeri M Mauri F Coupled dynamics of electrons and phonons in metallic nanotubes: current saturation from hot phonons generation Phys Rev B 2006 8 165419 1 6

[B16] Wang H Luo J Robertson A Ito Y Yan W Lang V Zaka M Schäffel F Rümmeli MH Briggs GAD Warner JH High-performance field effect transistors from solution processed carbon nanotubes ACS Nano 2010 8 6659 6664 10.1021/nn1020743 20958015

